# Burnout and predisposing factors in medical oncology in tunisia

**DOI:** 10.1192/j.eurpsy.2021.1151

**Published:** 2021-08-13

**Authors:** A. Daldoul, W. Khechin, W. Krir, F. Ezzairi, H. Kefi, S. Zaied, S. Ben Ahmed

**Affiliations:** 1 Medical Oncology, Fattouma Bourguiba University Hospital, Monastir, Tunisia; 2 Psychiatry, Military Hospital, Tunis, Tunisia; 3 Medical Oncology, University Hospital, sousse, Tunisia

**Keywords:** oncology, burn out

## Abstract

**Introduction:**

Health care professionals are particularly concerned with burnout

**Objectives:**

This study aimed to evaluate the the factors predisposing to occupational burnout

**Methods:**

This was a cross sectional study including health care professionals in medical oncology working in public hospitals in Tunisia. It was carried out from 15 January 2019 to 15 June 2019. Health profeessionals were asked to answer the Maslach –Burnout Inventory Test.

**Results:**

The mean age was 34 ± 6.7 years [23 - 57]. The sex ratio was 0.22. Our study population included 37 doctors (53%) and 33 nurses (47%). The inappropriate working conditions mentioned by the participants were as follows: The requirementss of patients and their families (91.5%), the lack of resources (87%), overwork found (83%), unsatisfactory effort- salary ratio (83%) and the reduced number of staff (77%). Several Burn-out factors mentioned by the participants were significantly associated with a high emotional exhaustion syndrome: overwork, poor service organization, lack of resources, lack of time, lack of recognition, conflicts with colleagues, lack of communication, unsatisfactory salary - effort ratio, assaults by patients. Several factors were positively and significantly associated with a high depersonalization score: overwork, poor service organization, small number of staff, lack of resources, lack of respect, lack of recognition. The global burnout associating the achievement of the three dimensions was significantly associated with overwork, lack of recognition, conflicts with colleagues and assault by patients.

**Conclusions:**

Burnout has become a major issue in Tunisian medicine. If left untreated, burnout epidemic may continue to worsen, to the detriment of patients and doctors

